# Pear-Shaped Craniopharyngioma: Selection of the Transcranial Approach With Respect to Topographical Classifications

**DOI:** 10.7759/cureus.64431

**Published:** 2024-07-12

**Authors:** Kemal Dizdarević, Mashhour A Alsuwat, Sara S Alrubie, Faisal S Alrubaei

**Affiliations:** 1 Department of Neurosurgery, King Abdulaziz Specialist Hospital, Taif City, SAU; 2 Department of Neurosurgery, University of Sarajevo, Sarajevo, BIH; 3 College of Medicine, Taif University, Taif City, SAU; 4 Department of Neurosurgery, King Faisal Medical Complex, Taif City, SAU

**Keywords:** suprasellar tumor, approach selection, pear shaped, lateral supraorbital approach, craniopharyngioma topography

## Abstract

We present the selection of the transcranial microsurgical approach, operative treatment, and outcomes following the resection of a pear-shaped craniopharyngioma (CP). A nine-year-old boy was operated on and followed up for 2.5 years after radical resection of the extrapial CP. Postoperatively, there was no tumor recurrence. The surgical strategy was discussed based on the preoperative MRI appearance of the CP, especially its morphological characteristics, including not only its size and shape but also its relationship with the hypothalamus, pituitary stalk/gland, ventricles, and optic chiasm, and the possible location of perforators. A description of the tumor topography is provided together with a discussion on the rationale for the selection of our surgical approach. Based on an understanding of the tumor topography, important information can be gained for approach selection, surgical planning, and anticipation of the hypothalamic-pituitary outcome.

## Introduction

Craniopharyngioma (CP) was earlier considered a benign intracranial extrinsic tumor [1]. Now, it is often classified as a low-grade malignancy that shortens life expectancy. A CP often arises from the pituitary stalk and involves the hypothalamus, suprasellar region, surrounding neurovascular structures, and third and lateral ventricles [2]. The squamous epithelial cell resting in Rathke’s cleft is the origin of the craniopharyngioma. It accounts for 7% of pediatric [3] and 3% of all intracranial tumors [4]. Recurrence could be expected after subtotal resection [1-5]. The correct decision about the surgical approach has an important impact on the outcome [1,2,5,6]. Based on the topography and morphology of the craniopharyngioma seen by preoperative MRI, the point of original tumor growth can be appreciated. Based on this, craniopharyngiomas can be classified roughly as infradiaphragmatic (intrasellar) and supradiaphragmatic (suprasellar) [1,5,7]. Here, we only discuss the relationship between transcranial approaches and the suprasellar craniopharyngioma topography; we are aware that the endonasal approach recently became an effective and dominant way of surgery not only for managing intrasellar lesions but also for lesions extending beyond the sella [7-9]. We want to stress the usefulness of the transcranial approach with reference to not only the intra-arachnoidal, suprasellar tumor location but also to emphasize the importance of the hypothalamus and the floor of the third ventricle during CP surgery. The operative dissection of CPs that originate from the upper part of the pituitary stalk and those that extend to the third ventricle from the suprasellar space seems to be easier and safer with the transcranial route [1,5,7,10,11]. A pear-shaped craniopharyngioma is a typical example of the suprasellar origin of the tumor pushing the diaphragm and pituitary gland downward. Suprasellar craniopharyngiomas could be additionally divided into three groups according to their relationship with the floor of the third ventricle: extraventricular, intra-extraventricular, and intraventricular. The direction and extent of the tumor can be comprehended only if we take into consideration both the vertical and the horizontal growth patterns [11]. The selection of the lateral supraorbital approach (LSO) [12] as a minimally invasive transcranial microsurgical route contributed to satisfactory outcomes in this case of suprasellar, extrapial, and purely extraventricular craniopharyngioma.

## Case presentation

A nine-year-old boy with complete visual loss, endocrine deficiencies, long-term cognitive impairment, and seizures controlled with three antiepileptic drugs (valproic acid, levetiracetam, topiramate) harbored craniopharyngioma and was operated on by the transcranial route.

**Figure 1:**
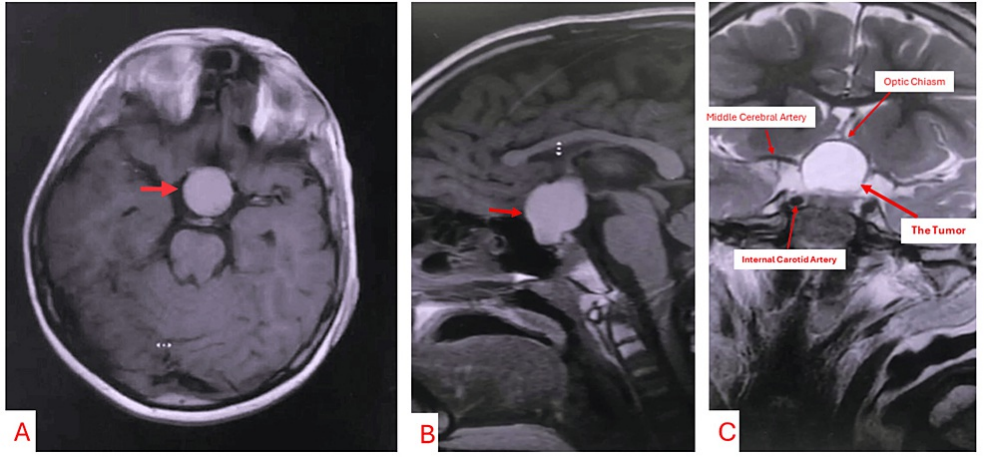
1a. T1 MRI of the brain in the axial plane at the level of rostral pons. The arrow points to the cystic, hyperintense part of the tumor. Hyperintensity reflects proteinaceous fluid. 1b. T1 MRI of the brain in the sagittal plane. The arrow points to the pear-shaped tumor, which is in a suprasellar-extraventricular location. No imaging signs of hydrocephalus. 1c. T2 MRI of the brain in the coronal plane. Significant distension of optic chiasm by the tumor. The chiasm is compressed from below and visible as a thin line over the superior surface of the tumor.

**Figure 2:**
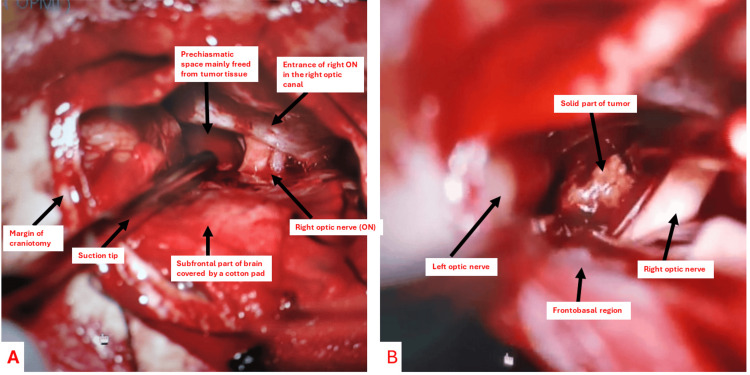
a, b) Intraoperative view The solid part of the prechiasmatic craniopharyngioma is visible in front of the chiasm and medially to the optic nerves. The post-fixed chiasm is covered by the frontobasal part of the brain.

MRI revealed the preoperative pear-shaped tumor appearance (Figure 1) congruent with a supradiaphragmatic tumor origin. A prechiasmatic tumor with postfixed chiasm was located intra-arachnoidally (Figures 2a, 2b intraoperative), meaning it had started to grow from the pituitary stalk (suprasellar extraventricular). The high signal of fluid seen on T1 is related to high protein content (Figure 1a, preoperative axial view). The signal on the T2 MRI was mainly bright, expressing a significant cystic component of the tumor (Figure 1c, preoperative coronal T2). Excessive extension of the patient’s optic apparatus by the tumor (Figure 1c) resulted in blindness. Microsurgical radical resection was done through a minimally invasive, right-sided lateral supraorbital approach, which is a modification of the classical pterional and subfrontal approaches (Figure 2b intraoperative).

**Figure 3:**
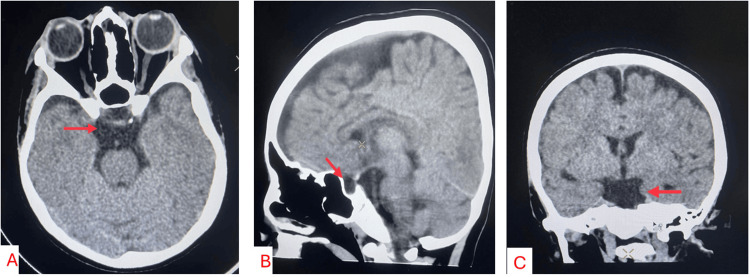
a, b, c) Axial, sagittal, and coronal views: CT scan performed 10 days after surgery; arrow demonstrates radical tumor removal

The patient was discharged 10 days after surgery with minimal improvement of vision and in good general condition. Radical tumor removal was shown by a CT scan performed before discharge (Figures 3a-3c).

**Figure 4:**
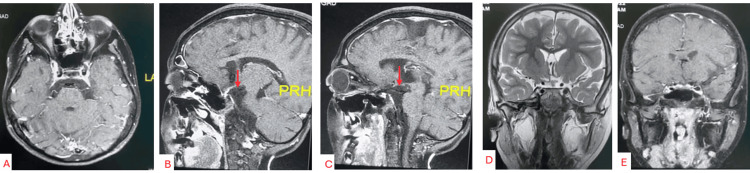
a, b, c, d, e) Axial, sagittal, and coronal planes, postoperative MRI with contrast showing complete tumor resection with intact floor of the third ventricle as exhibited in b, c

Radical resection without recurrence was confirmed by postoperative serial MRIs with and without contrast during the follow-up period (Figures 4-4e).

There was no postoperative focal neurological deficit and no diabetes insipidus. The electrolyte balance was normal. Levothyroxine (25 mcg/daily) and cortisol (25 +15 mg/daily) replacement therapy was administrated.

Regular follow-up over 2.5 years showed no tumor recurrence. There are no additional delayed neurological deficits or endocrinological deficiencies. The patient has been receiving antiepileptic monotherapy (levetiracetam) after surgery. Visual impairment slightly improved but without functional vision.

## Discussion

Craniopharyngiomas starting to grow from the pituitary stalk are located intraarachoidally and extrapially in the suprasellar cisterns and grow below the chiasm and the floor of the third ventricle so their location is supradiaphragmatic but purely extraventricular. This kind of tumor extends anteriorly in front of the chiasm between optic nerves (prechiasmatic with a postfixed chiasm (Figures 2a-2b). Tumors with upper infundibulum origin can grow behind the chiasm between the optic tracts. This tumor can break the floor of the third ventricle so the lower part of the tumor grows suprasellary but the upper part extends into the third ventricle. Such tumors are intraventricular-extraventricular, intrapially located craniopharyngiomas and have direct contact with the hypothalamus. This location is not suitable for the endonasal approach. Infundibular CP can rarely be localized only in the third ventricle, meaning completely intraventricularly without involvement of the ventricular floor. The first valuable classification of CPs in the context of surgical approaches was done in 1990 by Yasargil [1]. It is based on the CP's relation to the diaphragma sellae, the tuber cinereum, and the hypothalamus. The selection of the approach should be determined by the relation between the chiasm and sella turcica. The chiasm could be prefixed, normal, and postfixed. A craniopharyngioma is considered pre-chiasmatic when it displaces the chiasm posteriorly (postfixed chiasm over the dorsum sella). This location of the tumor is best attacked through different modifications of subfrontal approaches.

Those CPs located behind the chiasm produce a prefixed chiasm so subfrontal approaches are not suitable due to interference with the central part of the optic apparatus, which is positioned over tuberculum sella and covers a significant part of the tumour.

Infradiaphragmatic CPs resected by transcranial approaches result in more hypopituitarism because it is easier to access the sella from below and to protect the pituitary gland using this route. The classical surgical attitude based on Yasargil’s experience with CPs implies that the pterional-transsylvian, transcallosal-interhemispheric, and transsphenoidal approaches can cover all topographic types of CPs [1]. As the pterional-transsylvian approach allows early identification of the stalk and optic apparatus as well as perforators from anterior circulation, suprasellar intra-arachnoidal CPs are suitable to be resected by this route. When CPs extend in the third ventricle, the combination of the pterional and transcallosal approaches can be used. The transcallosal approach is primarily reserved for pure intraventricular CPs and transsphenoidal approaches for intrasellar infradiaphragmatic tumors [1]. Fan et al. classified these tumors as “QST” types: subdiaphragmatic (type Q CP); subarachnoidal (type S-CP); and pars tuberalis (type T-CP) (7). The QST classification puts specific importance on the exact localization of CP origin and its relation to the hypothalamus-pituitary axis.

Q-CPs imply difficulty in tumor-pituitary surgical dissection so hormone deficiency is common. Contrarily, this type is clearly separated from the hypothalamus so dissection can be controlled even if CPs have significant suprasellar extension. A good postoperative hypothalamic status can be expected despite the less satisfactory endocrine outcome. Transphenoidal endonasal surgery is the primary surgical option for the majority of Q-CPs, except for those with extremely suprasellar extension.

S-CPs arise from the stalk and have a mainly suprasellar intra-arachnoid location but can extend into ventricles. The adhesion is commonly found between the tumor and the stalk. Preservation of the hypothalamus and pituitary is a reachable goal. According to Fan et al., the endonasal approach is usually superior for this type of tumor. Endonasal surgery could be indicated for S-CPs only if the tumor does not occupy numerous subarachnoid cisterns.

T-CPs are ventricular tumors with hypothalamic disturbances. The outcome is significantly better with microsurgery than with endoscopy. Craniotomy and microsurgery are strongly indicated for giant intraventricular tumors 

Our presented patient had the S-CP type of tumor. The transcranial, lateral supraorbital approach was selected (Figure 4) [12]. There are two main types of subfrontal approaches: minimally invasive (a. lateral supraorbital and b. eyebrow) and extensive (a. fronto-orbital with and b. without removal of the supraorbital ridge).

Generally, transcranial treatment of CP includes extraaxial and transaxial approaches [10].

The main extraaxial approaches are subfrontal and pterional [1,4,5,13,14] with many modifications [6,12]. The subfrontal approach can involve different trajectories (subchiasmatic, interhemispheric, transsphenoidal, lateral to the optic chiasm and tract, lateral to ICA, and lamina terminalis) [12,14,15]. It can also be modified by subfronto-orbital access performed with unilateral, one-and-a-half, or bilateral bone removal techniques [15]. Subfrontal interhemispheric implies the craniotomy extending to the frontal base with the deliberate opening of the frontal sinus. According to Fahlbusch et al., this is the best approach for resecting large CP especially those with retrochiasmatic and suprasellar extensions [2].

For some giant CPs, a subfronto-orbital approach with a large surgical entrance could be useful. It is preferable if CSF release and tumor cyst opening are difficult to achieve early in surgery. A typical example of this is a giant CP with a large calcified segment.

Yasargil’s pterional approach always includes the opening of the Sylvian fissure [1]. Levy et al [13] proposed the fronto-orbitozygomatic-temporopolar approach for pediatric CP. This complex surgery is a combination of zygomatic and temporopolar modifications of the pterional and subtemporal approaches.

A patient with giant intraventricular-extraventricular CP (Samii gr V) [14] could be operated on applying a bilateral, subfronto-orbital microsurgical approach, which includes frontal craniotomy with orbital osteotomy performed separately [10,15]. The supraorbital ridges and anterior two-thirds of the orbital roofs were removed, leaving the ethmoidal bone intact and preserving the olfactory nerves. This approach is extended by the transcranial transsphenoidal route. The sella is entered from the superior part of the sphenoid sinus, and a CP from the sella turcica can be resected easily. The sealing of the sella and sinus must be done before closure.

Hernesniemi’s LSO approach is very comfortable for suprasellar, intra-arachnoidal, extra-pial, pear-shaped, and mainly cystic CPs which originate from the lower and middle part of the stalk [12]. This topography of CP implies clear separation from the hypothalamus and pituitary gland even when the tumor involves multiple basal cisterns and has a considerable size. The LSO is a minimally invasive, fast-performing approach with short skin incision. The elevation of the skin-muscle flap as a single layer protects the frontotemporal branches of the KN VII. The temporalis muscle is preserved, reducing postoperative masticatory dysfunction. One burr hole followed by key-hole craniotomy, leaving the supraorbital ridge in place and straightforward access to the CSF release through lamina terminalis, enables the neurosurgeon to gain the optimal trajectory for a suprasellar CP attack, especially due to the almost symmetrical bilateral basal view of the neurovascular structure around tumors.

## Conclusions

The minimally invasive lateral supraorbital approach is suitable for the safe radical resection of suprasellar, subarachnoidal, extrapial, and pear-shaped craniopharyngiomas. The clear separation of this morphological type from the hypothalamus and the pituitary gland is a crucial topographical fact that enables neurosurgeons to achieve radicality of resection with minimal morbidity.
